# A nomogram to predict large-for-gestational-age in term newborns: A retrospective single-center study

**DOI:** 10.1097/MD.0000000000046580

**Published:** 2025-12-19

**Authors:** Yingyun Wu, Jianting Ma

**Affiliations:** a Department of Obstetrics and Gynecology, The Affiliated Yangming Hospital of Ningbo University (Yuyao People’s Hospital of Zhejiang Province), Yuyao, China.

**Keywords:** large-for-gestational-age, newborn, nomogram, risk factor

## Abstract

This study aims to build and validate a nomogram for large-for-gestational-age (LGA) prediction in full-term (37–41 weeks of gestation) newborns. This retrospective single-center study included consecutive full-term deliveries (37–41 weeks’ gestation) at Yuyao People’s Hospital of Zhejiang Province from January to December 2021. Participants were randomly assigned (7:3) to training and validation sets. The current study included 1481 deliveries (training n = 1017; validation n = 464). Gestational age (odds ratio [OR] = 1.32, 95% confidence interval [CI]: 1.13–1.54), gestational weight gain (OR = 1.07, 95% CI: 1.03–1.12), symphysis-fundal height (OR = 1.14, 95% CI: 1.03–1.27), fetal abdominal circumference (OR = 1.10, 95% CI: 1.09–1.12), triglycerides (OR = 1.49, 95% CI: 1.08–2.05), gestational diabetes mellitus (OR = 2.05, 95% CI: 1.33–3.14), and pre-pregnancy body mass index < 18.5 (OR = 0.48, 95% CI: 0.28–0.81) had independent associations with LGA. A nomogram was developed, and receiver operating characteristic curves had areas under the curves of 0.846 (95% CI: 0.821–0.871) and 0.802 (95% CI: 0.761–0.842) in the training and validation sets, respectively. Seven factors were independently associated with LGA. A nomogram was developed and showed favorable predictive performance.

## 1. Introduction

Large-for-gestational-age (LGA) denotes infants born with abnormally high weights.^[1,2]^ A major risk factor for LGA is elevated maternal body mass index (BMI),^[3]^ but the relationship varies among populations. LGA is mostly related to BMI in African–American women, while age is a major contributor to LGA in Asians.^[3]^ High pregestational BMI, excessive gestational weight gain (GWG), and impaired glucose tolerance are linked to LGA in Asians.^[4]^ In Caucasians, excessive GWG and glycated hemoglobin levels are associated with LGA.^[5]^ Improvements in economic development and dietary diversity over the past decades have been paralleled by an increased incidence of over-nutrition during pregnancy, leading to elevated LGA incidence.^[1,2]^ LGA has significant associations with adverse perinatal outcomes and increased long-term risks of death, hospitalization, obesity, hypertension, and type 2 diabetes mellitus.^[1,2]^

An issue is that the risk of LGA must be evaluated early to make appropriate decisions for the mother and the infant. Unfortunately, current screening approaches for LGA have poor effectiveness. A first-trimester model based on multiparity, pregestational BMI, and plasma protein A (PAPP-A) amounts had 55% sensitivity and 79% specificity.^[6]^ Another first-trimester model for LGA considering nuchal translucency, β-human chorionic gonadotrophin, and PAPP-A also had moderate accuracy.^[7]^ Other models are limited to mothers with obesity^[8]^ or diabetes,^[9]^ lacking generalizability. Mid-pregnancy models based on amniotic fluid examination (which is invasive)^[10]^ and ultrasound^[11]^ display relatively good accuracy. In addition, PAPP-A quantitation is not available or performed everywhere. Therefore, simple-to-use models applicable to all women and using routine parameters are needed for LGA early screening.

Therefore, the current work aimed to develop and validate a nomogram predictive model for LGA in full-term newborns.

## 2. Materials and methods

### 2.1. Study design and participants

The present retrospective analysis examined consecutive pregnant women at Yuyao People’s Hospital of Zhejiang Province between January 2021 and December 2021. The inclusion criterion was pregnant women with singleton pregnancies who delivered at full term (Gestational age [GA] of 37–41 weeks). Exclusion criteria were: severe comorbidities or obstetric complications, including type 1 or 2 diabetes, chronic hypertension, pregestational dyslipidemia, cardiovascular disease, acute or chronic liver or kidney diseases, or severe diseases of other organs; treatment with medications affecting glucose or lipid metabolism; or incomplete data. The protocol was approved by the Ethics Committee of Yuyao People’s Hospital of Zhejiang Province (Approval No. 2021KY1073). The requirement for informed consent was waived owing to the retrospective design.

### 2.2. Data collection and definitions

The clinical information and laboratory test results were collected from the maternal and child electronic monitoring system and our hospital’s electronic medical record system. All exclusion-related conditions (type 1/2 diabetes, chronic hypertension, pregestational dyslipidemia, cardiovascular, hepatic, and renal disorders) were confirmed by standardized examinations performed at our hospital before or during the first prenatal visit. Laboratory indicators were measured in the hospital’s central laboratory using standardized methods. No data were based on patient self-report. Variables collected included maternal age, pregestational BMI, numbers of pregnancies and deliveries, conception method, adverse maternal history (i.e., fetal loss, pre-eclampsia, or premature birth), and GWG.

GWG was calculated using the weight measured at 12 weeks of gestation as baseline and the weight recorded at admission for delivery as the follow-up value. Pre-pregnancy BMI categories (underweight, <18.5 kg/m²; normal weight, 18.5–24.9 kg/m²; overweight, 25.0–27.9 kg/m²; and obesity, ≥28 kg/m²) were defined according to the Guidelines for Pre-pregnancy and Pregnancy Care (2018)^[12]^ and the Principles for Adult Weight Management (2024 edition),^[13]^ which recommend a BMI cutoff of 28 kg/m² for obesity in the Chinese population. These criteria differ from the WHO classification and are widely used in Chinese obstetric research. Symphysis fundal height was measured beginning at the 20th week of pregnancy, and the value at admission was used for analysis. Fetal AC was measured by ultrasound at admission for delivery, with a time allowance of ±3 days.

Additional variables included complications during gestation (hypertensive disorders of pregnancy and gestational diabetes mellitus [GDM]), late pregnancy blood lipids and other metabolic indicators (total cholesterol, triglycerides [TG], high-density lipoprotein cholesterol and low-density lipoprotein cholesterol, free fatty acids [FFA; reflecting maternal lipid metabolism and insulin resistance], total bile acids [used in the screening of intrahepatic cholestasis of pregnancy], and homocysteine [Hcy; a biomarker of methylation metabolism and vascular health]), delivery GA, and neonatal birth weight. LGA was defined as birthweight above the 90th percentile for GA,^[1]^ consistent with standard definitions, and is commonly associated with maternal risk factors such as dyslipidemia during pregnancy, obesity, and diabetes.

Obstetric care at our institution followed unified clinical pathways and standard operating procedures based on national obstetric quality-management standards. Symphysis-fundal height was measured at admission by trained midwives using a non-elastic tape from the superior border of the symphysis pubis to the uterine fundus with the participant in the supine position. Fetal AC was obtained by experienced sonographers using uniform machine settings and a standardized fetal biometry protocol. All biochemical indicators were assayed in the hospital’s central laboratory on calibrated analyzers under internal quality control. These procedures were implemented to minimize inter-operator variability.

### 2.3. Statistical analysis

SPSS 26.0 (SPSS) and Stata 17.0 (Stata Corporation, College Station) were employed for data analysis. Normally and skewedly continuous variates were presented as mean ± standard deviation and median with interquartile range (P25-P75, IQR), respectively, and compared by the Student *t* test and Wilcoxon’s rank-sum test, respectively. Categorical variables, reported as n (%), were compared by the chi-square test. Two-tailed *P* < .05 was considered statistically significant.

The required sample size was estimated using the 1-in-10 rule (events per variable), which suggests that at least 10 outcome events are needed for each independent variable. With 19 covariates, a minimum of 190 events was required, and the present dataset included 534 LGA cases, which was sufficient. Participants were then randomly assigned into training and validation sets in a 7:3 ratio using SPSS. Univariate and multivariable logistic regression analyses were conducted using the training set to construct the prediction model. Variables with *P* < .10 in univariate analysis were included in multivariable logistic regression. GWG was analyzed as a continuous variable and pre-pregnancy BMI category was included as a covariate; interaction between GWG and BMI category was not modeled in the present nomogram. Based on the multivariate analysis results in the training set, a nomogram was generated with the Stata package nomolog. Scores for individual variables were obtained from regression coefficients and added up to obtain total scores for various patients, which may be converted into predicted probabilities of LGA occurrence. Model differentiation and calibration were assessed by receiver operating characteristic and calibration curve analyses. No probability threshold was prespecified. Because sensitivity and specificity fluctuate with the chosen cutoff, they were not used as primary performance metrics. Instead, model performance was summarized by AUC, with calibration curves, the Hosmer–Lemeshow test, and decision curve analysis (DCA) to evaluate calibration and clinical utility. The net benefit of each model was examined by DCA. External validation of the model was performed in the validation set using discrimination, calibration, and DCA.

## 3. Results

Totally 1481 women were assessed, including 1017 and 464 in the training and validation sets, respectively. Mean birth weight was 3316.69 g. LGA newborns accounted for 36.06% (534/1481) of all deliveries, with a mean birth weight of 3745.61 ± 227.25 g (Table 1).

**Table 1:** Maternal characteristics.

Characteristic	Training set (n = 1017)	Validation set (n = 464)	*P*
Age (yr)	29.31 ± 4.44	29.44 ± 4.53	.600
GA (wk)	38.94 ± 1.1	39.01 ± 1.10	.230
TC (mmol/L)	6.03 ± 1.13	6.056 ± 1.31	.760
LDL-C (mmol/L)	3.10 ± 0.92	3.15 ± 1.02	.370
HDL-C (mmol/L)	1.82 ± 0.39	1.83 ± 0.43	.480
GWG (kg)	13.67 ± 4.07	13.83 ± 3.85	.480
SFH (cm)	32.95 ± 1.81	32.89 ± 1.73	.530
Fetal AC (mm)	334.61 ± 14.65	335.43 ± 13.52	.300
Number of pregnancies	2 (1, 3)	2 (1, 3)	.570
Number of deliveries	0 (0, 1)	0 (0, 1)	.150
TG (mmol/L)	2.94 (2.24, 3.89)	2.85 (2.16, 3.77)	.270
FFA (mmol/L)	0.37 (0.27, 0.54)	0.37 (0.26, 0.52)	.200
TBA (μmol/L)	2.5 (1.8, 3.85)	2.8 (1.8, 3.9)	.330
Hcy (μmol/L)	6.37 (5.51, 7.5)	6.42 (5.58, 7.51)	.340
Mode of conception			
Natural pregnancy	975 (95.90%)	450 (97.00%)	.300
Assisted reproduction	42 (4.10%)	14 (3.00%)	
Adverse maternal history			.320
Yes	107 (10.50%)	41 (8.80%)	
None	910 (89.50%)	423 (91.20%)	
HDP			.090
Yes	130 (12.80%)	45 (9.70%)	
None	887 (87.20%)	419 (90.30%)	
GDM			.920
Yes	184 (18.10%)	83 (17.90%)	
None	833 (81.90%)	381 (82.10%)	
Pre-pregnancy BMI (kg/m^2^)			.040
<18.5	153 (15.00%)	67 (14.40%)	
18.5–24.9	713 (70.10%)	352 (75.90%)	
25.0–27.9	91 (8.90%)	30 (6.50%)	
≥28.0	60 (5.90%)	15 (3.20%)	
LGA			.440
Without	657 (64.60%)	290 (62.50%)	
With	360 (35.40%)	174 (37.50%)	

Data are mean ± standard deviation, median (P25, P75), or n (%). 0 cell (0.0%) had an expected count of <5.

AC = abdominal circumference, BMI = body mass index, FFA = free fatty acids, GA = gestational age, GDM = gestational diabetes mellitus, GWG = gestational weight gain, Hcy = homocysteine, HDL-C = high-density lipoprotein cholesterol, HDP = hypertensive disorders of pregnancy, LDL-C = low-density lipoprotein cholesterol, LGA = large-for-gestational-age, SFH = symphysis fundal height, TBA = total bile acids, TC = total cholesterol, TG = triglycerides.

The multivariable logistic regression analysis revealed GA (odds ratio [OR] = 1.32, 95% confidence interval [CI]: 1.13–1.54, *P* < .01), GWG (OR = 1.07, 95% CI: 1.03–1.12, *P* < .01), symphysis-fundal height (SFH; OR = 1.14, 95% CI: 1.03–1.27, *P* = .01), abdominal circumference (AC; OR = 1.1, 95% CI: 1.09–1.12, *P* < .01), TG (OR = 1.49, 95% CI: 1.08–2.05, *P* = .02), GDM (OR = 2.05, 95% CI: 1.33–3.14, *P* < .01), and pre-pregnancy BMI < 18.5 kg/m^2^ (OR = 0.48, 95% CI: 0.28–0.81, *P* = .01; Table 2) had independent associations with LGA.

**Table 2:** Univariable and multivariable logistic regression analyses.

Training set (n = 1017)	Univariable logistic regression analysis		Multivariable logistic regression analysis	
	OR (95% CI)	*P*	OR (95% CI)	*P*
Age (yr)	1.02 (0.99, 1.05)	.140		
GA (wk)	1.57 (1.38, 1.77)	<.010*	1.32 (1.13, 1.54)	**<.001**
TC (mmol/L)	0.94 (0.83, 1.05)	.250		
LDL-C (mmol/L)	0.87 (0.75, 1.00)	.050*		
HDL-C (mmol/L)	0.62 (0.44, 0.86)	<.010*		
GWG (kg)	1.11 (1.07, 1.14)	<.010*	1.07 (1.03, 1.12)	**<.001**
SFH (cm)	1.43 (1.32, 1.56)	<.010*	1.14 (1.03, 1.27)	**.010**
Fetal AC (mm)	1.12 (1.10, 1.13)	<.010*	1.1 (1.09, 1.12)	**<.001**
Number of pregnancies	1.01 (0.77, 1.32)	.960		
Number of deliveries	1.36 (1.05, 1.76)	.020*		
TG (mmol/L)	1.56 (1.20, 2.02)	<.010*	1.49 (1.08, 2.05)	**.020**
FFA (mmol/L)	0.92 (0.71, 1.19)	.530		
TBA (μmol/L)	0.91 (0.70, 1.17)	.460		
Hcy (μmol/L)	1.06 (0.82, 1.37)	.670		
Mode of conception	0.64 (0.32, 1.28)	.210		
Adverse maternal history	1.15 (0.76, 1.74)	.510		
HDP	0.92 (0.63, 1.36)	.690		
GDM	1.70 (1.23, 2.34)	<.010*	2.05 (1.33, 3.14)	**<.001**
Pre-pregnancy BMI (kg/m^2^)				
18.5–24.9	Ref.	–	–	–
<18.5	0.37 (0.24, 0.57)	<.010*	0.48 (0.28, 0.81)	**.010**
25.0–27.9	1.23 (0.79, 1.91)	.370	1.06 (0.61, 1.85)	.830
≥28	1.96 (1.15, 3.32)	.010	1.32 (0.65, 2.7)	.440

Bold indicates that the results reached statistical significance at the .05 level.

AC = abdominal circumference, BMI = body mass index, CI = confidence interval, FFA = free fatty acids, GA = gestational age, GDM = gestational diabetes mellitus, GWG = gestational weight gain, Hcy = homocysteine, HDL-C = high-density lipoprotein cholesterol, HDP = hypertensive disorders of pregnancy, LDL-C = low-density lipoprotein cholesterol, OR = odds ratio, SFH = symphysis fundal height, TBA = total bile acids, TC = total cholesterol, TG = triglycerides.

* Variables with *P* <.10 in univariate analysis were included in multivariable logistic regression.

**Figure 1.**
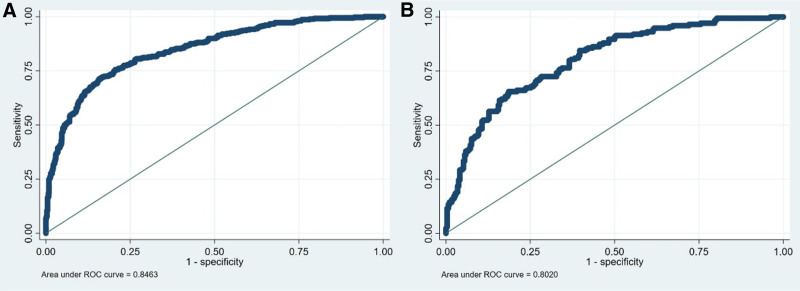
ROC curves. (A) The training set. (B) The validation set. ROC = receiver operating characteristic.

**Figure 2.**
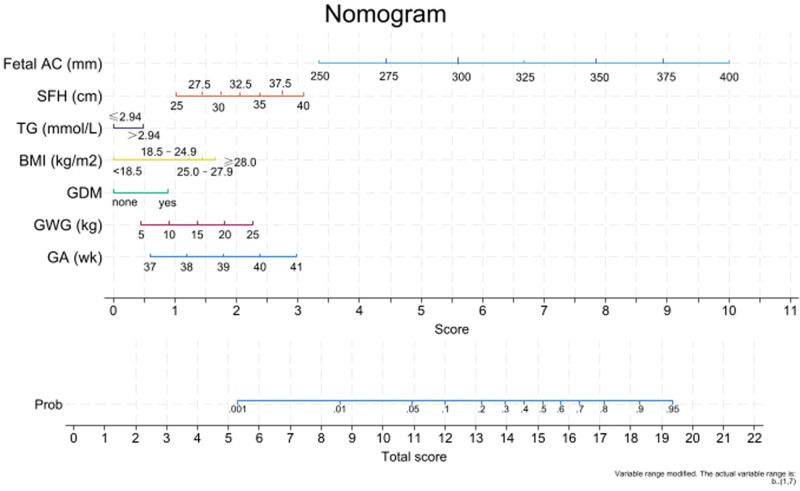
Nomogram. AC = abdominal circumference, BMI = body mass index, GA = gestational age, GDM = gestational diabetes mellitus, GWG = gestational weight gain, SFH = symphysis fundal height, TG = triglycerides.

Receiver operating characteristic curve analysis of the combination of the 7 factors showed areas under the curves of 0.846 (95% CI: 0.821–0.871) and 0.802 (95% CI: 0.761–0.842) in the training and validation sets, respectively (Fig. 1). A nomogram was developed (Fig. 2). The Hosmer–Lemeshow test (Fig. S1, Supplemental Digital Content, https://links.lww.com/MD/Q965) and DCA (Fig. S2, Supplemental Digital Content, https://links.lww.com/MD/Q965) yielded goodness-of-fit and a net clinical benefit, indicating good predictive performance for the nomogram. The higher the total score, the higher the predicted probability of LGA (Fig. 2). To use the nomogram, locate each predictor on its axis (pre-pregnancy BMI, GWG, GDM, TG, GA at delivery, SFH at admission, and fetal AC at admission), assign points for each, sum the points, and project the total to the “Predicted probability of LGA” scale to obtain the individualized risk estimate.

## 4. Discussion

This study developed a nomogram based on 7 factors independently associated with LGA, which showed favorable predictive performance. The nomogram model might help clinicians assess the birth weight to identify the optimal mode of delivery and decrease the incidence of adverse pregnancy outcomes.

Mounting evidence suggests pre-pregnancy obesity, GWG, and GDM are risk factors for LGA in newborns.^[1,2]^ Still, the predictive abilities of these 3 factors are suboptimal, but no study has hitherto successfully developed an early prediction model for LGA. This study established and validated a predictive model for LGA newborns. The results showed that GA, GWG, SFH, fetal AC, TG, GDM, and pre-pregnancy BMI were independent predictors of LGA newborns. Of note, all those variables are readily available as part of the maternal clinical workup, in opposition to some models using tests that are not available everywhere (e.g., PAPP-A).^[6,7]^ In addition, some models have been developed specifically for mothers with obesity^[8]^ or diabetes,^[9]^ lacking applicability to all pregnant women. In the present study, the model demonstrated good discrimination in both the training and validation sets (AUC 0.846 and 0.802, respectively) and acceptable calibration and net benefit, comparing favorably with previously reported first-trimester models.^[6,7]^

The present study showed that pre-pregnancy BMI was an independent predictor of LGA. With an increase in BMI, birth weight also increases. Obesity has been identified as a major health problem in pregnancy, affecting nearly 25% of pregnant women.^[1,2]^ Current evidence suggests that higher rates of maternal obesity do not necessarily result in elevated offspring birth weights, and obese mothers with good metabolic profiles have offspring with lower birth weights (−94 [95% CI: −150 to −38] g per 1 standard deviation (6.5%) of obese mothers with good metabolism, *P* = .001).^[14]^ Pre-pregnancy basal body mass can influence neonatal weight, and lifestyle needs to be considered to increase metabolism during pregnancy.

The increase in GWG or body mass was found to independently predict LGA and was included in the prediction model, causing a 1.07-fold elevation of LGA risk for each 1 kg of weight gain in the gestation period, with a GWG cutoff value of 14.25 kg, consistent with findings by Song and colleagues.^[15]^ A systematic evaluation and meta-analysis of >1 million pregnant women reported 47% with GWG that exceeded the ACOG recommendations.^[16]^ Analyzing the birth certificate data of >5,00,000 women in Michigan and New Jersey, USA, a study reported a significant relationship between GWG and birth weight^[17]^ (β = 7.35 [95% CI: 7.10–7.59], *P* < .0001), with women with GWG > 24 kg giving birth to newborns 148.9 g heavier (95% CI: 141.7–156.0) than those with a GWG of 8 to 10 kg and 2.26 times higher risk of giving birth to a newborn >4000 g. Another study on patterns of GWG suggested that excessive weight gain during early gestation could be associated with fetal overgrowth, whether later weight gain occurs or not.^[18]^ The above reports and the Domestic Expert Consensus on Exercise in Pregnancy (Draft)^[19]^ recommend adequate exercise during gestation to control weight gain and reduce complications. Future studies on predictive models should examine weight gain during gestation and consider weight control at different stages and individualized formulation of weight gain to help adjust interventions reducing the incidence of LGA newborns. Indeed, even though the present study showed that the incremental increase of 1 kg in GWG was associated with LGA, a 1-kg increase in GWG probably does not have the same meaning in a 20-kg/m^2^ woman compared with a 40-kg/m^2^ woman since pre-pregnancy BMI is known to influence the maternal, perinatal, and fetal outcomes.^[20]^ Further model refinement is necessary. For clinical interpretation, recommended total GWG ranges differ by pre-pregnancy BMI (approximately 12.5–18 kg for underweight, 11.5–16 kg for normal weight, 7–11.5 kg for overweight, and 5–9 kg for obesity), and the GWG component of the nomogram should be interpreted in that context.^[21]^

The present study suggested that LGA incidence was 2.05-fold higher in women with GDM versus women without GDM. A meta-analysis showed that the risk of LGA was 1.81-fold higher in GDM women compared with the non-GDM group.^[16]^ Shen et al^[22]^ found that a single fasting-glucose measurement was comparable to a 75-g oral glucose tolerance test in predicting LGA risk, but a study in Japan^[23]^ showed that 75-g oral glucose tolerance test results were not associated with LGA occurrence. The national guidelines for the diagnosis and management of hyperglycemia in pregnancy^[24]^ recommends screening to be performed at pregnancy weeks 24 to 28. A recent report suggested the association of GDM with LGA starts at pregnancy week 20 and becomes significant at week 28.^[25]^ Accordingly, interventions to mitigate GDM-induced fetal overgrowth should be made prior to pregnancy week 24 to 28. Further investigation is warranted to examine whether the timing of screening should be advanced for more effective intervention.^[26]^

Moreover, this study showed that TG is an independent predictor of LGA, with TG levels >2.91 mmol/L reflecting a 1.49-fold elevation in LGA’s probability. The increase in TG is often overlooked due to altered lipid metabolism during gestation and the fact that fatty acids constitute an important energy source besides glucose for fetal growth, especially in the second trimester, and are essential for adequate retinal and neurological development.^[27]^ In women with GDM, higher postprandial 2-h FFA levels (non-fasting FFA) in mid-gestation increase LGA risk.^[28]^ Besides maternal glucose, TG and FFA are also crucial for fetal fat accumulation. Harmon et al^[29]^ suggested that glucose does not exhibit the strongest correlation with infant body fat percentage, while TG measured early (*r* = 0.67, *P* < .001) and FFAs measured late in pregnancy (*r* = 0.54, *P* < .01) have stronger correlations. Therefore, attention should be paid to the structure of the diet at the end of pregnancy to prevent TG from exceeding 2.91 mmol if possible.

In the present study, including GA as a clinical parameter increased the accuracy of neonatal birth weight prediction. Within the full-term window (37–41 weeks), later GA at delivery was independently associated with higher odds of LGA. Because LGA status was defined using gestational-age-specific birth-weight percentiles, this association reflects an adjusted cohort effect near term rather than GA alone determining LGA. SFH and AC are internationally recognized as the most convenient and acceptable methods for assessing LGA newborns. A sum of both parameters reaching or surpassing 140 cm suggests the possibility of LGA.^[30]^ The present study demonstrated for every 1 cm elevation in SFH after term, LGA’s risk increased by 1.14-fold, with a uterine height cutoff value of 32.47 cm. Among ultrasound parameters, fetal weight estimated from AC measured before delivery was considered the best predictor of LGA in a previous report.^[31]^ Ultrasound screening during late gestation can increase the diagnostic rate of LGA.^[32]^ Recently, a systematic evaluation and meta-analysis reported AC > 360 mm exhibited good performance in predicting LGA with a sensitivity of >50% (positive likelihood ratio of 7.56, 95% CI: 5.85–9.77).^[33]^

Nevertheless, there may be several limitations. First, early pregnancy data to predict the probability of LGA for primary prevention during early pregnancy were lacking. Hence, some of the variables included in the nomogram are measured when LGA is already in place and can be detected by ultrasound. Future studies should focus exclusively on pre-pregnancy and first-trimester parameters. In the meantime, the nomogram could be used during discussions about C-section delivery. Given that this was a retrospective analysis, the accuracy of our findings may be affected to a certain extent by documentation and reporting biases. In addition, the data analyzed were limited to those found in patient charts. Scores previously shown to be associated with LGA could not be calculated retrospectively.^[34–37]^ The cohort was drawn from a single tertiary hospital in an economically developed coastal city in eastern China, which may limit generalizability to other parts of the province or country. Maternal demographics, BMI distributions, nutritional patterns, and obstetric practices may differ across inland and rural settings and among regions with different ethnic composition. External validation across diverse settings is warranted; if systematic differences are observed, model transport may require recalibration of the intercept and slope rather than redevelopment. There was also a lack of data on other environmental and maternal characteristics that may affect birth weight, for example, diet, environment, smoking, genetics, and ethnicity. Furthermore, information on genetic or syndromic causes of overgrowth, such as Beckwith-Wiedemann, Sotos, or Costello syndromes, was not available and therefore such conditions could not be excluded. Finally, we did not stratify the GWG effect by BMI category nor test a GWG × BMI interaction, and future work will evaluate BMI-specific GWG effects. Although care pathways and measurement standard operating procedures were unified within the institution, we did not quantify inter-operator variability; any residual non-differential measurement error would be expected to bias associations toward the null rather than create spurious effects.

In conclusion, a nomogram based on 7 factors independently associated with LGA was developed and showed favorable predictive performance. The nomogram can provide clinicians with a simple and intuitive tool to detect and identify LGA newborns, intervene in early pregnancy, and adopt a rational mode of delivery to avoid adverse maternal and infant outcomes. The model will be continuously optimized in the future.

## Acknowledgments

All the authors thank the support and assistance provided by the Affiliated Yangming Hospital of Ningbo University.

## Author contributions

**Conceptualization:** Yingyun Wu, Jianting Ma.

**Data curation:** Yingyun Wu.

**Formal analysis:** Yingyun Wu.

**Writing – original draft:** Yingyun Wu, Jianting Ma.

**Writing – review & editing:** Yingyun Wu, Jianting Ma.

## Supplementary Material


