# Supplementary material for: A nomogram to predict large-for-gestational-age in term newborns: A retrospective single-center study
Source: Medicine (Baltimore). 2025 Dec 19;104(51):e46580. doi: 10.1097/MD.0000000000046580 (PMC12727380; doi:10.1097/MD.0000000000046580)

**Supplementary digital content Figure S1.** Calibration curves. (A) The training set. (B) The validation set.

E:O, The ratio of expected to observed events; CITL, calibration in the large; AUC, area under the ROC curve; CI, confidence interval.


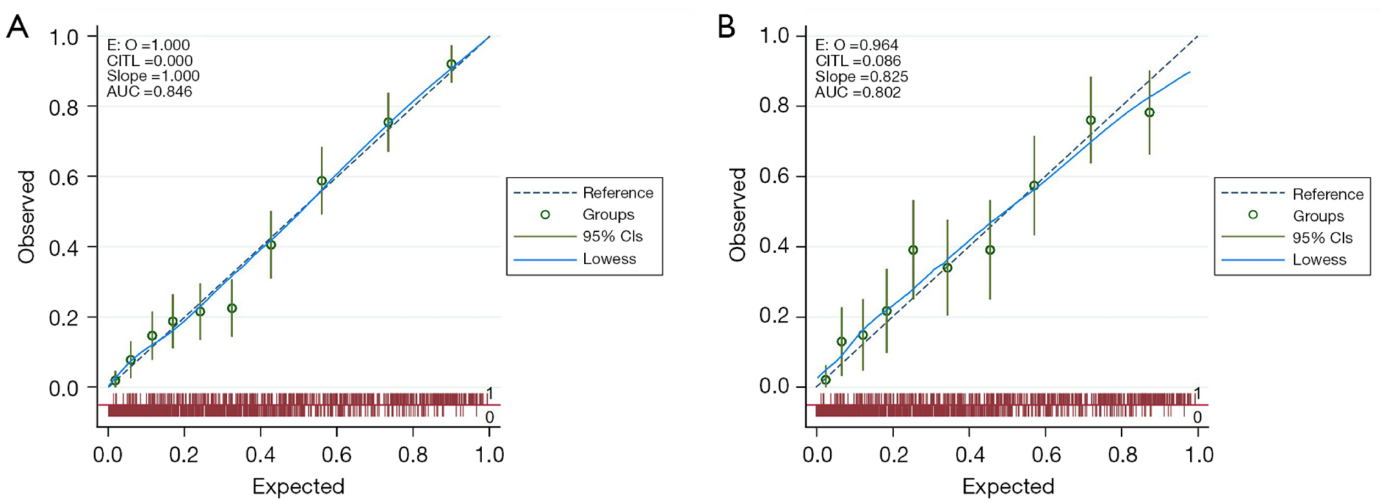


**Supplementary digital content Figure S2.** DCA. (A) The training set. (B) The validation set.

DCA, decision curve analysis.


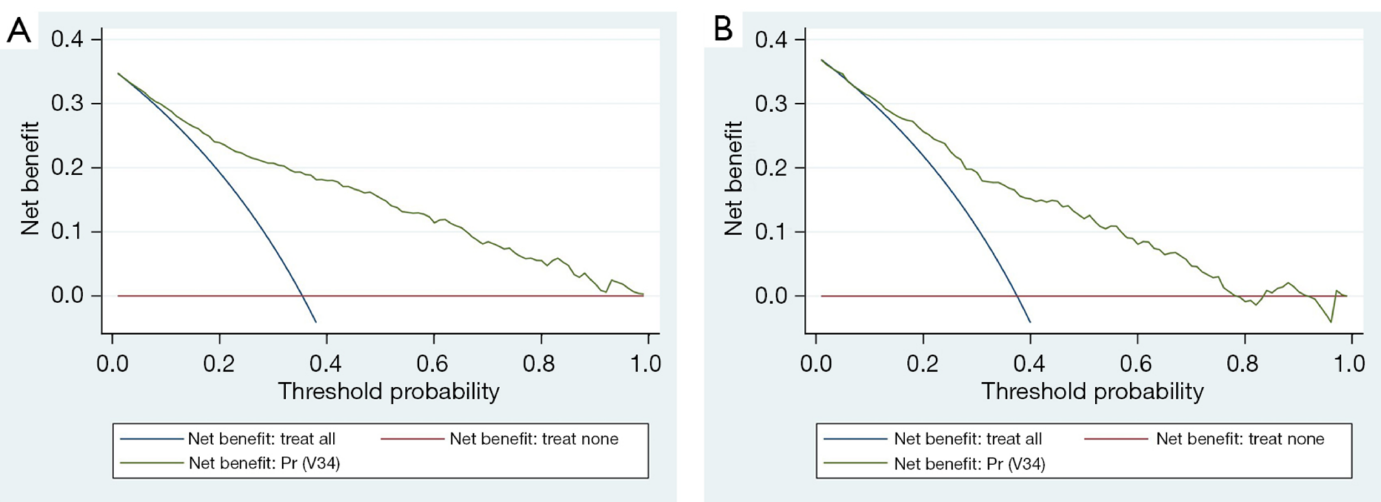

Supplement: Supplementary file 1 [file medi-104-e46580-s001.docx]
